# Fecal biomarkers of environmental enteric dysfunction and associated factors among children aged 24–59 months in east Dembiya district, northwest Ethiopia

**DOI:** 10.1186/s12876-022-02255-4

**Published:** 2022-04-08

**Authors:** Zemichael Gizaw, Alemayehu Worku Yalew, Bikes Destaw Bitew, Jiyoung Lee, Michael Bisesi

**Affiliations:** 1 Department of Environmental and Occupational Health and Safety, Institute of Public Health, College of Medicine and Health Sciences, University of Gondar, Gondar, Ethiopia; 2 Addis Continental Institute of Public Health, Addis Ababa, Ethiopia; 3 Global One Health Initiative (GOHi), The Ohio State University, Columbus, OH USA; 4 School of Public Health, Addis Ababa University, Addis Ababa, Ethiopia; 5 Division of Environmental Health Sciences, College of Public Health, The Ohio State University, 1841 Neil Avenue, Columbus, OH 43210 USA; 6 Department of Food Science and Technology, The Ohio State University, Columbus, OH USA

**Keywords:** Environmental enteric dysfunction, Fecal biomarkers, Gut inflammation, Enzyme-linked immunosorbent assay, Children, East Dembiya district, Ethiopia

## Abstract

**Background:**

Environmental enteric dysfunction is a subclinical intestinal disorder characterized by gut inflammation accompanied by morphological changes, such as blunted villi and crypt hyperplasia. This is a common illness in low and middle-income countries. However, environmental enteric dysfunction evidence is limited in Ethiopia. Accordingly, this study was conducted to measure fecal biomarkers of environmental enteric dysfunction and associated factors among children aged 24–59 months in rural northwest Ethiopia.

**Methods:**

A community-based cross-sectional study was employed among 235 randomly selected children in a rural setting of the east Dembiya district. Stool samples were collected without fixative and analyzed for fecal biomarkers of environmental enteric dysfunction (Alpha-1-antitrypsin, neopterin, and myeloperoxidase) using commercial enzyme-linked immunosorbent assay kits and analyzed for intestinal parasites using wet mount and Kato-Katz techniques. Child behaviors related with exposure to enteropathogens, condition of the living environment and socio-demographic information were collected using interviewer-administered questionnaire and structure observation. We fitted multivariable linear regression model to assess the association between environmental factors and concentration of fecal biomarkers of environmental enteric dysfunction in the stool. Statistically significant associations were declared based on adjusted betas with the corresponding 95% confidence interval and *p*-value < 0.05.

**Results:**

The median concentration of fecal markers of environmental enteric dysfunction was 350 μg/ml for Alpha-1-antitrypsin, 3320.2 ng/ml for myeloperoxidase, and 1562 nmol/l for neopterin. The median concentration of Alpha-1-antitrypsin among 161 (68.5%), myeloperoxidase among 168 (71.5%), and neopterin among 188 (80%) of the stool samples were above the normal values in non-tropical settings. Moreover, 100 (42.6%) of the children had high EED disease activity score (above the median score). The elevated concentrations of fecal biomarkers of gut inflammation and the high EED disease activity score were significantly associated with open defecation practice, mouthing of soil contaminated materials, *Escherichia coli (E. coli) * contamination of drinking water, *E. coli* contamination of foods, *E. coli* contamination of soil, and intestinal parasites.

**Conclusion:**

Overall, Alpha-1-antitrypsin, myeloperoxidase, and neopterin levels among the children in the studied region were highly elevated in comparison to populations in high-income countries. Moreover, the EED disease activity score in significant proportion of children was high, suggesting widespread intestinal inflammation and increased intestinal permeability. Extensive *E. coli* contamination of the living environment (drinking water, ready-to-eat foods, and courtyard soil), hygiene and sanitation behaviors (such as open defecation and mouthing of soil contaminated materials), and a high burden of intestinal parasites were identified as factors associated with the elevated concentration of fecal biomarkers of environmental enteric dysfunction. Parental care to children to avoid mouthing of soil contaminated materials and other risky behaviors that increase exposure enteric infections, and protecting the living environment (water, food and soil) from fecal contamination are important.

## Background

Environmental enteric dysfunction (EED) is a subclinical intestinal disorder, formerly known as tropical enteropathy, was first observed in adults from low and middle income countries (LMICs) in the 1960s, when an abnormal microscopic appearance of the small bowel was observed. The villi were found to be blunted and shortened, resulting in a smaller surface area for nutrient absorption. In the late 2000s, tropical enteropathy was renamed environmental enteropathy (EE) to reflect emerging evidence that the quality of the environment was more important than climate or latitude. It has been renamed EED in recent years [1, 2]. Environmental enteric dysfunction is distinguished by gut inflammation as well as morphological changes such as blunted villi and crypt hyperplasia [3].

The geographic distribution of EED suggests that EED is most prevalent in areas of poor access to improved water and sanitation. In addition, biomarkers of EED have been strongly associated with storage of fecal matter near households and unimproved water sources in LMICs [4–6]. For instance, the MAL-ED multi-country study measured fecal concentration of Alpha-1-antitrypsin, (AAT), myeloperoxidase (MPO), and neopterin (NEO) in LMICs as biomarkers of EED and reported that children in LMICs, such as children in Bangladesh, India, Nepal, Pakistan, Peru, Brazil, Tanzania, and South Africa had elevated concentrations of fecal biomarkers of EED [7] compared with concentration considered normal to populations in high-income countries, i.e., < 270 μg/ml for AAT, < 2000 ng/ml for MPO, and < 70 nmol/l for NEO [7, 8].

Environmental enteric dysfunction has multiple causal pathways. Repeated exposure to enteropathogens is a well-documented cause in the current literature. People living in areas with poor sanitation are constantly exposed to enteropathogens that cause enteric infections through the consumption of contaminated water, foods, and in some cases, soil [3, 9, 10]. The ingested enteropathogens may cause chronic inflammation. Exposure to chemical toxicants such as pesticides, drugs, and aflatoxins are also cause intestinal damage including increased leakiness of the gut barrier [11, 12].

Environmental enteric dysfunction can be diagnosed by endoscopic assessment, biomarkers, nonradioactive stable isotope assays, and -omics technologies [13]. The assessment of biopsies from intestinal biopsy and histological analysis is considered to be the gold standard to assess EED. This procedure, however, is deemed overly invasive, difficult, and costly [14, 15]. Stable isotope techniques to diagnose EED involves oral administration of an isotopically labeled compound and subsequent monitoring of the appearance of the compound or its catabolic products in breath, feces, urine, and/or blood [13]. But, this has been limited by expense and analytical difficulties. Moreover, despite the wide commercial availability of stable isotope labelled compounds, official guidelines may not be established in developing countries and the preparations have not, been cleared for clinical use [16, 17]. Different -omics technologies (such as genomics, epigenomics, transcriptomics, proteomics, and metabolomics) can be used to diagnose EED [13]. However, developing countries may not have access to these technologies and may not have skilled manpower and other resources.

In recent years, a number of potential non-invasive biomarkers of EED have become available [18, 19]. The lactulose: mannitol (L:M) dual sugar test [20, 21] and fecal biomarkers of gut inflammation (namely, AAT, MPO, and NEO) [7] are the most widely used non-invasive biomarkers to diagnose EED. Oral administration of lactulose and mannitol sugars is followed by a timed urine collection in the L:M dual sugar test [20, 21]. Lactulose is big enough to only pass through the paracellular leak pathway or epithelial damage sites. Mannitol, which is three times smaller, crosses the pore channel and can be considered as a measure that combines surface area and exposure time [21–23]. The L: M ratio determines how permeable the leaky channel and epithelium barrier are. Increased L: M values indicate a problem with the gut. However, it is not standardized and is technically difficult for children because it needs fasting and urine collection for up to five hours prior to testing [21].

Fecal AAT, MPO, and NEO have been, therefore, widely used as stool biomarkers of gut inflammation to diagnose EED [18]. Because AAT is not synthesized in the intestine, its presence in the stool indicates protein loss, increased blood-to-intestinal permeability, and gut inflammation [24]. Myeloperoxidase is an enzyme active during infection to kill microbes [25] and NEO is released during pro-inflammatory responses [26]. A combination of biomarkers can better explain EED than any single biomarker and it is documented that EED disease activity score can be calculated using fecal AAT, MPO, and NEO [7, 27].

In EED, the gut architecture is disrupted, and tight connections between cells are broken, resulting in a porous intestine that can allow bacteria or bacterial products to enter the systemic circulation [20]. This could lead to immunological activation and a systemic inflammatory state, with negative health consequences. Translocation-induced acute phase proteins, for example, have been demonstrated to inhibit insulin-like growth factor 1 (IGF-1) and cause growth hormone resistance [28]. This could impact linear growth [18, 29], cognitive development, immunological responses to pathogens [18, 30], and even later life obesity, diabetes, and metabolic syndrome [6]. Additionally, systemic inflammation has been linked to decreased vaccination efficacy [31].

Environmental enteric dysfunction is a poorly understood condition that may have far-reaching impacts on child growth, health, and development in LMICs [32]. It is now the subject of significant research interest as investigators seek to define its causes, pathogenesis, consequences, and possible preventive approaches. No evidence is available in Ethiopian context about EED and its predisposing factors. Accordingly, this study was conducted to assess fecal biomarkers of EED and associated factors among children aged 24–59 months in rural northwest Ethiopia.

## Methods

### Study design, setting, and period

This community-based cross-sectional study was conducted in a rural setting of the east Dembiya district of Ethiopia from 01 May to 18 June 2021. The east Dembiya district is one of the districts in central Gondar zone, the Amhara national regional state. As of July 2020, the district had a total of 192,020 rural and 18,741 urban residents [33], of these, 39,927 (12.22%) were children under age 5-years [34]. In the district, coverage of clean water and latrine were 26.6% and 55%, respectively. Moreover, intestinal parasitic infections and diarrheal diseases were the top four and five prevalent diseases, which accounted 5161 (9.97%) and 4981 (9.62%), respectively [35].

### Sample size calculation and sampling procedures

Sample size was calculated using single population proportion formula with the following assumptions: proportion of children with elevated concentration of fecal MPO and AAT = 82% [8], level of significance (α) = 5%, 95% confidence interval, margin of error (d) = 5%, and a non-response rate of 5%. Therefore, the sample size (n) = 238. All households with children aged between 24 and 59 months in the rural kebeles (the lowest administrative unit in Ethiopia) in the district were considered for sampling. First, we chose six rural kebeles at random out of 28 kebeles using a simple random sampling technique. We allocated equal number of households to each kebele. Finally, 238 households with children aged 24–59 months were included in the study using a systematic random sampling technique. Children below 24 months old were excluded from the study since it is difficult to get stool samples from younger children. Moreover, children with severe clinical illness, who need inpatient nutritional therapy, and who received deworming and antibiotics in recent time prior to this study period were excluded.

### Environmental sample collection

Water, food, and soil samples were collected aseptically. To collect stored water, sample collectors, who are Environmental Health experts asked mothers to provide a glass of water from their primary drinking water storage container, as if they were giving it to their children, and pour 100 ml into a sterilized sampling bottle [36]. To collect soil samples, the respondents were asked to identify the outdoor area where the youngest child aged 24–59 months had most recently spent time and sample collectors then took approximately 50 g of soil using a sterile scoop and plastic bag [36]. Sample collectors asked mothers to provide approximately 2 g of food in the same manner they feed their children and scooped the whole portion to fill a sterile plastic bag using a sterile spoon [36]. Moreover, sample collectors observed child behaviors that would result in hand or mouth contact with environmental fomites (mouthing of soil or soil contaminated materials, such as objects or foods on the ground, eating dirt, mouthing hands, etc.) for 30 min spot observation. The water, food, and soil samples were stored in ice box for transportation and preserved at 4 °C in the laboratory until analyzed for fecal indicator bacteria (i.e., *E. coli*).

### Stool sample collection

Stool sample collectors, who are Environmental Health experts or mothers first told children to urinate first without pooping to avoid urine contamination of the stool. To avoid stool contamination with soil or dirt, stool sample collectors or mothers told children to defecate on a paper. Stool sample collectors used wooden stick to transfer approximately 50 g of the last part of the stool, the softest part, into the collection container after the child defecated on the paper. Stool sample collectors then immediately delivered the sample to the stool examination team, who stationed at the center of the village where stool samples were collected in order to facilitate fresh stool analysis. After investigation of ova of parasites, stool samples were stored in ice box for transportation and preserved at − 20 °C in the laboratory for a maximum of three weeks until analyzed for fecal biomarkers of EED.

### Detection of *E. coli* in water, food and soil samples

1 g of food and soil samples were homogenized with a sterile peptone-buffered water (PBW, 0.1%) (10 ml for food and 20 ml for soil) using a sterile blending bag and a laboratory-scale processor for 1 min at the specified mixing speed. Serial dilutions were done using sterile distilled water by tenfold dilution. 10 ml of solution from 10^−4^ to 10^–3^ dilutions were taken. The water samples were not diluted before being analyzed. The entire water sample, soil, and food solutions were separately filtered through a 47-mm diameter, 0.45-µm pore-sized sterile filter membrane (Millipore, Burlington, MA, USA) and cultured on membrane lauryl sulphate broth pouring into an absorbent pad (Oxoid Limited, Basingstoke, UK). The prepared samples were incubated for 24 h at 44.5 °C before counting the number of colony forming units (CFU) according to the standard procedures outlined in the WHO guideline [37]. The filtration apparatus was washed with distilled water and flamed between analyses of consecutive samples and sterilized at intervals. The colony number was counted and the results were expressed as CFU per 100 ml of water or 1 g of soil and food samples by taking into consideration of dilution factors. One field blank per sample collectors per week, plus one laboratory blank per laboratory assistants per day were processed for quality control.

### Detection of ova of parasites in stool samples

Ova of intestinal parasites in stool samples were detected using direct stool examination (wet mount) and Kato-Katz techniques. Stool specimens were diluted with saline as necessary for direct examination. 0.05 g of stool specimen was placed, mixed with a drop of saline, and covered with a cover slide. Finally, the specimen was examined under the microscope at low (×10 objective) and high (×40 objective) magnification powers for the identification of intestinal parasites [38]. A small amount of feces (approximately 2 g) was placed on a scrap piece of paper for the Kato-Katz. Using applicator stick, the stool was pressed against the top of the fecal specimen's screen. The template was placed on a clean microscopic slide and filled with the sieved fecal specimen after the upper surface of the screen was scraped to sieve the fecal specimen. The template was then carefully removed, leaving the entire fecal specimen on the slide. The fecal specimen that remained was covered with a glycerol-soaked cellophane strip and examined under a ×10 objective microscope [38].

### Measurement of biomarkers

We used commercial ELISA kits obtained from USA to measure stool concentrations of MPO (Immundiagnostik AG, Germany), AAT (BioVendor, ImmuChorm, Germany), and NEO (GenWay Biotech Inc., USA) in the Gondar Blood Bank Lab, Gondar, Ethiopia. All protocols were followed as per manufacturers’ instructions, which are included in the kits. Commercial standards and controls were run in duplicate for each run to monitor assay performance and reliability. The final dilution of fecal biomarkers was determined by selecting the most appropriate concentration of a biomarker falling in the linear range of standard curve. Myeloperoxidase was performed in two dilutions of 1:50 initial dilution and 1:10 s dilution, NEO at the dilution of 1:500 and AAT at an initial dilution of 1:50 and second dilution of 1:250. Samples out of range of the standard curve for any of the assays were run at a twofold higher or lower concentration (as appropriate) [7]. All plates were read on HumaReader HS plate reader (Human, Germany).

We used the 4-parameter algorithm since it gives the best standard curve fit to calculate concentration of fecal biomarkers to each sample. We first plotted the optical density (OD) values of standards (y-axis) against their concentration (x-axis). We then read the concentration of the samples directly from the standard curve using CurveExpert 1.3 ELISA data analysis software. Concentration of biomarker values were examined for outliers, normality and compared with values considered normal in non-tropical settings: AAT < 270 μg/ml, MPO < 2000 ng/ml, and NEO < 70 nmol/l [7, 8].

### Measurement of study variables

Environmental enteric dysfunction, the primary outcome variable of the study was measured by three stool fecal biomarkers, namely MPO, AAT, and NEO. Fecal biomarker concentrations were categorized based on the distribution of all measurements: low (in first quartile), medium (in the interquartile range), or high (in fourth quartile). For each of the three biomarkers, 0 point was given for low concentrations in first quartile, 1 point for medium concentrations in the interquartile range, and 2 points for high concentrations in fourth quartile. The EED disease activity score was, then, calculated as 2 × (AAT category) + 2 × (MPO category) + 1 × (NEO category). The EED disease activity score can range from 0 (lowest quartile in all categories) to 10 points (highest quartile in all categories) [7, 39, 40].

Mouthing of soil contaminated materials, fecal contamination of drinking water, fecal contamination of ready-to-eat foods, fecal contamination of courtyard soil, and intestinal parasites were the exposure variables for this study. Childhood diarrheal disease was defined as having three or more loose or watery stools within 24 h period [41]. A two-week period diarrheal disease in children was determined based on history from mothers or caregivers and a 24 h diarrheal disease in children was determined by looking the nature of the stool. Prevalence of intestinal parasites in children was defined as the presence of one or more ova of intestinal parasitic species in stool samples [42]. Drinking water, ready-to-eat foods, and courtyard soil were taken as fecally contaminated if* E. coli*, the most common indicator organism of fecal contamination, was found in water, food, and soil samples [43]. Geophagy is the intentional recurring or repeated ingestion of nonfood substances (such as clays, yard soil), or large quantities of certain particular foods contaminated with soil [44].

### Statistical analysis

Stata version 14 (Stata Corp, College Station, TX, USA) was used to analyze the data. Spearman linear correlations were calculated between concentrations in AAT, MPO, and NEO in stool. Multivariable linear regression analysis was done to assess the association between environmental factors and concentration for gut inflammation biomarkers in the stool. Socio-economic factors such as age and livestock ownership were also entered into the models to control confounding effect. Covariates for the adjusted model were selected using bivariate analysis on the basis of *p*-values < 0.2. In the adjusted model, statistically significant associations were declared based on adjusted betas with the corresponding 95% confidence interval (CI) and *p*-value < 0.05.

## Results

### Socio-demographic characteristics and environmental conditions

The summary statistics about the children included in this study and the sanitation conditions of their living environment is presented in Table 1. One hundred and sixty-six (70.6%) of the households with children aged between 24 and 59 months owned livestock. One hundred and sixty-eight (71.5%) of the water samples at point of use were positive with *E. coli*. Furthermore, 160 (68.1%) of the food samples and 193 (82.1%) of the courtyard soils were also positive with *E. coli*. During the 30 min spot check observation, we observed that 172 (73.2%) of the children mouthed soil contaminated materials (Table 1).

**Table 1:** Socio-demographic characteristics of study participants and sanitation conditions of the living environment in rural northwest Ethiopia, May to June 2021, (n = 235)

Socio-demographic and sanitation conditions	Frequency	Percentage
Sex of children		
Male	114	48.5
Female	121	51.5
Age of children in month		
24–36	54	23.0
37–48	78	33.2
49–59	103	43.8
Livestock ownership		
Yes	166	70.6
No	69	29.4
*E. coli* detected in drinking water		
Yes	168	71.5
No	67	28.5
*E. coli* detected in ready-to-eat foods		
Yes	160	68.1
No	75	31.9
*E. coli* detected in courtyard soil		
Yes	193	82.1
No	42	17.9
Mouthing of soil contaminated materials		
Yes	172	73.2
No	63	26.8

### Diarrheal disease and intestinal parasites in children

One or more intestinal parasitic species were identified in 145 (61.7%) of the stool samples, out of which 35 (14.9%) of the stool samples had multiple parasites. We confirmed that 31 (13.2%) of the children had diarrheal disease at the time of the survey and 74 (31.5%) of mothers or care givers reported that their child had diarrhea in a 2-week period prior to the survey. Twenty-five (10.6%) of the children had both diarrheal disease and intestinal parasites (Table 2).

**Table 2:** Prevalence of intestinal parasites and diarrheal disease among children in rural northwest Ethiopia, May to June 2021, (n = 235)

Health conditions	Frequency	Percent
Intestinal parasites		
No	90	38.3
Single parasite	110	46.8
Multiple parasites	35	14.9
Diarrheal disease in the last 24 h		
Yes	31	13.2
No	204	86.8
Diarrhea in the last two weeks		
Yes	74	31.5
No	161	68.5

### Fecal biomarkers of environmental enteric dysfunction

The median concentration of fecal biomarkers of EED was 350 μg/ml for AAT, 3320.2 ng/ml for MPO, and 1562 nmol/l for NEO. Significant spearman linear correlation coefficients were found between AAT and MPO (0.45), AAT and NEO (0.46), and MPO and NEO (0.43). The median concentration of AAT among 161 (68.5%), MPO among 168 (71.5%), and NEO among 188 (80%) of the stool samples were above the normal values in non-tropical settings (Table 3).

**Table 3:** Concentration of fecal biomarker of EED in stool samples collected from children aged between 24 to 59 months in rural northwest Ethiopia, May to June 2021, (n = 235)

Biomarkers	Median (25th, 75th percentile)	Children with elevated fecal biomarkers, n (%)	Spearman linear correlation coefficients		
			AAT	MPO	NEO
AAT (μg/ml)	350 (268, 385)	161 (68.5)	1.00		
MPO (ng/ml)	3320.2 (1890, 6528.2)	168 (71.5)	0.45	1.00	
NEO (nmol/l)	1562 (450, 1852)	188 (80)	0.46	0.43	1.00

Table 4 shows the EED disease activity score of children. The median (with 25th and 75th percentile) EED disease activity score was 5 (3, 7). Results depicted that 100 (42.6%) of the children had high EED disease activity score (above the median score), indicating that the concentrations of fecal biomarkers in these children are elevated.

**Table 4:** EED disease activity score of children aged between 24 and 59 months in rural northwest Ethiopia, May to June 2021, (n = 235)

EED disease activity score	Frequency	Percent
0	21	8.9
1	8	3.4
2	22	9.4
3	19	8.1
4	21	8.9
5	44	18.7
6	19	8.1
7	34	14.5
8	26	11.1
9	13	5.5
10	8	3.4

### The association between environmental conditions and fecal biomarkers of EED

After adjusting for age of children, concentration of fecal biomarkers of EED and EED disease activity score were significantly associated with defecation practice of households, mouthing of soil contaminated materials, *E. coli* contamination of drinking water, *E. coli* contamination of ready-to-eat foods, *E. coli* contamination of courtyard soil, and intestinal parasites (Table 5).

**Table 5:** Associations of environmental conditions and fecal biomarkers of EED in children aged 24–59 months in rural northwest Ethiopia, May to June 2021

Variables	AAT	MPO	NEO	EED
	Adjusted beta (95% CI)	Adjusted beta (95% CI)	Adjusted beta (95% CI)	Adjusted beta (95% CI)
Age of children				
24–35 months	–	Reference	Reference	Reference
36–47 months	–	417.86 (− 319.76, 1155.47)	127.80 (− 78.31, 333.91)	− 0.78 (− 1.41, − 0.15)*
48–59 months	–	714.00 (80.92, 1347.08)*	− 195.81 (− 372.53, − 19.09)*	− 1.267 (− 1.88, − 0.66)***
Households defecation practice				
Open field	4.85 (− 8.21, 17.92)	884.80 (265.25, 1504.36)**	91.55 (− 81.61, 264.71)	0.71 (0.20, 1.23)**
Sanitary latrine	Reference	Reference	Reference	Reference
Mouthing of soil contaminated materials				
Yes	− 12.58 (− 32.73, 7.56)	1236.34 (275.32, 2197.35)*	383.79 (117.63, 649.96)**	0.97 (0.18, 1.76)*
No	Reference	Reference	Reference	Reference
*E. coli* recovered in soil				
Yes	25.20 (4.25, 46.15)*	375.20 (− 618.00, 1370.39)	396.02 (119.98, 672.06)**	1.00 (0.18, 1.82)*
No	Reference	Reference	Reference	Reference
*E. coli* recovered in water				
Yes	5.27 (− 11.88, 22.42)	1865.46 (1035.41, 2695.51)***	151.71 (− 79.68, 383.10)	1.53 (0.85, 2.22)***
No	Reference	Reference	Reference	Reference
*E. coli* recovered in food				
Yes	4.44 (− 10.76, 19.63)	758.50 (26.31, 1490.70)*	133.86 (− 70.51, 338.23)	0.45 (− 0.16, 1.06)
No	Reference	Reference	Reference	Reference
Intestinal parasites				
No	Reference	Reference	Reference	Reference
Single parasites	80.03 (62.00, 98.07)***	85.94 (− 768.95, 940.84)	236.39 (− 2.41, 475.18)	1.40 (0.69, 2.11)***
Multiple parasites	106.09 (82.91, 129.27)***	1564.85 (445.57, 2684.12)**	363.86 (52.07, 675.65)*	2.69 (1.79, 3.61)***

^*^Statistically significant at *p* < 0.05, **Statistically significant at *p* < 0.01, ***Statistically significant at *p* < 0.001, - *p* > 0.2 in the bivariate analysis

The bacteriological quality of courtyard soil was statistically associated with concentration of AAT in stool samples. The concentration of fecal AAT was increased by 25.20 μg/ml among children who lived in areas where the courtyard soil was contaminated with *E. coli* (β: 25.20, 95% CI (4.25, 46.15). Moreover, the concentration of AAT was increased by 80.03 μg/ml in children with single intestinal parasite (β: 80.03, 95% CI (62.00, 98.07) and multiple parasites explained an increase of 106.09 μg/ml of AAT concentration in stool samples (β: 106.09, 95% CI (82.91, 129.27) (Table 5).

This study revealed that high level of fecal MPO in children was associated with mouthing of soil contaminated materials. The concentration of fecal MPO was increased by 1236.34 ng/ml among children who mouthed soil contaminated materials compared with children without mouthing of soil contaminated materials (β: 1236.34, 95% CI (275.32, 2197.35). The concentration of fecal MPO was also higher among children whose families practiced open defecation compared with children whose families had utilized latrine (β: 884.80, 95% CI (265.25, 1504.36). *Escherichia coli* presence in drinking water predicted an increase of 1865.46 ng/ml concentration of fecal MPO (β: 1865.46, 95% CI (1035.41, 2695.51). *Escherichia coli *contamination of ready-to-eat foods resulted in high concentration of fecal MPO in children (β: 758.50, 95% CI (26.31, 1490.70). The concentration of fecal MPO was increased by 1564.85 ng/ml among children who had multiple intestinal parasites (β: 1564.85, 95% CI (445.57, 2684.12) (Table 5).

An elevated concentration of fecal NEO was statistically associated with mouthing of soil contaminated materials, *E. coli* contamination of courtyard soil, and intestinal parasites. The concentration of NEO in stool was increased by 383.79 nmol/l among children who mouthed soil contaminated materials compared with children who did not mouth soil contaminated materials (β: 383.79**,** 95% CI (117.63, 649.96). *Escherichia coli* contamination of courtyard soil was also associated with high concentration of fecal NEO in children (β: 396.02, 95% CI (119.98, 672.06). Moreover, intestinal parasites in children resulted an elevated concentration of fecal NEO (β: 363.86, 95% CI (52.07, 675.65) (Table 5).

The composite EED disease activity score was associated with indiscriminate disposal of human feces. The composite EED disease activity score was increased by 0.71 point in children in areas where open defecation is commonly practiced (β: 0.71, 95% CI (0.20, 1.23). The composite EED disease activity score was increased by 0.97 point among children who mouthed soil contaminated materials (β: 0.97, 95% CI (0.18, 1.76). The composite EED disease activity score was significantly associated with *E. coli* contamination of courtyard soil (β: 1.00, 95% CI (0.18, 1.82) and *E. coli* contamination of drinking water (β: 1.53, 95% CI (0.85, 2.22). Intestinal parasites in children explained a higher point increase in EED composite score. Moreover, the composite EED disease activity score was increased by 1.40 points in children with single intestinal parasite (β: 1.40, 95% CI (0.69, 2.11) and by 2.69 points in children with multiple intestinal parasites (β: 2.69, 95% CI (1.79, 3.61) (Table 5).

## Discussion

This community-based cross-sectional study was conducted to measure fecal biomarkers of EED among children aged 24–59 months in rural northwest Ethiopia. The median concentration of AAT in 68.5%, MPO in 71.5%, and NEO in 80% of the children were highly elevated in comparison with concentrations considered normal to populations in high-income countries [7, 8]. Moreover, 42.6% of the children had high EED composite score, i.e., above the median score, implies that these children had elevated concentration of AAT, MPO, and NEO. The elevated concentrations of fecal biomarkers of EED and the high EED disease activity score might be explained by poor water, sanitation and hygiene (WASH) conditions and high burden of enteric infections among children in the area. As explained elsewhere, the targeted children in the studied region were lived in extensively contaminated environment and in areas where different intestinal parasites are heavily presented and transmitted [45]. The elevated concentrations of fecal biomarkers of EED may imply repeated exposures of children to enteric infections, chronic gut inflammation, and morphological changes in the small intestine, increased permeability, and malabsorption of nutrients [3, 12]. However, although these biomarkers permit assessment of intestinal/systemic inflammation and/or intestinal epithelial barrier dysfunction, the main limitation to their use is that they are not specific for EED because they correlate with prevalence, activity, and/or severity of various other gastrointestinal diseases [13].

The concentrations of fecal biomarkers of EED and composite EED disease activity score were elevated among children who lived in households practicing open defecation. This finding is supported by findings of other studies [4, 46]. This is due to the fact that human feces contains dozens of disease-causing microorganisms [47, 48], which are the underline causes of EED. Utilization of sanitary latrine can break the chain of infection transmission from excreta of infected persons and can prevent the development of EED [48–50].

This study revealed that concentrations of fecal biomarkers of EED (MPO and NEO) and the composite EED disease activity score were associated with mouthing of soil contaminated materials. Children who mouthed soil contaminated materials had elevated concentration of fecal MPO and NEO and increased composite EED disease activity score compared with children without mouthing of soil contaminated materials. Similarly, concentrations of AAT, NEO and EED composite score were high among children who lived in areas where the courtyard soil was contaminated with *E. coli.* These findings are in agreement with findings of another study [51]. The effect of mouthing of soil contaminated materials and *E. coli* contamination of soil can be explained that the courtyard soil is heavily contaminated with fecal materials in developing countries where human and animals share the same living environment and open defecation is common. Mouthing of soil contaminated materials plays a greater role in the development and transmission of enteric infections [40, 52]. As it is well documented, exposure to enteropathogens is the leading cause of EED or elevated concentration of fecal biomarkers of EED [53–55].

This study documented that potential fecal contamination of drinking water and ready-to-eat foods measured by *E. coli* were significantly associated with elevated concentration of fecal biomarkers of EED and high EED composite score in children. This finding is in line with findings of other studies [56–59]. When food and water are fecally contaminated, children continuously exposed to enteropathogens through the ingestion of contaminated foods and water [3, 12, 60, 61]. The ingested enteropathogens may result in the chronic gut inflammation accompanied by morphological changes, such as blunted villi, and crypt hyperplasia [3, 12].

Children with intestinal parasites had elevated biomarkers of EED and EED composite disease activity score. Infections with enteropathogens can increase gut inflammation and permeability and may result in systemic inflammation [20]. Multiple physiological mechanisms by which enteropathogens can disrupt gut functioning have been identified [62–64]. Some microorganisms like rotavirus, adenovirus, and astrovirus cause limited mucosal disturbances. Others, such as campylobacter, shigella, salmonella, enteroaggregative E. coli (EAEC), enteropathogenic *E. coli* (EPEC), and enteroinvasive *E. coli* (EIEC) are enteroinvasive or cause extensive mucosal disruption. Others like enterotoxigenic *E. coli* (ETEC) cause of secretory diarrhea with only limited mucosal changes [20].

Our study had some important limitations. Despite the fact that EED is explained by various enteropathogens as discussed above, we only examined the presence of various intestinal parasites in children. The use of EED biomarker values from high-income countries to define cut-off limits for normal or elevated biomarker concentration is another limitation. This is not a unique limitation to this study. Others have pointed out a lack of reference values for EED biomarkers in children living in low-income households [8, 65]. There is thus a need for large-scale studies to investigate enteropathogens associated with EED and to establish reference values for EED biomarkers in children living in low income settings.

## Conclusion

Overall, AAT, MPO, and NEO levels among the children in the study area located in Ethiopia were highly elevated in comparison to populations in high-income countries. Moreover, the EED disease activity score in significant proportion of children was high, suggesting widespread intestinal inflammation and increased intestinal permeability. Open defecation practice, mouthing of soil materials, fecal contamination of the living environment (water, foods, and soil), and intestinal parasites were associated with the elevated concentration of fecal biomarkers and the high EED disease activity score. Parental care to children to avoid mouthing of soil materials, promoting latrine utilization, protecting the home environment (water, food and soil) from fecal contamination, and avoiding risky behaviors that increase exposure to intestinal parasites are important.

## Data Availability

Data will be made available upon requesting the primary author.

## Abbreviations

μg/ml: Microgram per milliliter
AAT: Alpha-1-antitrypsin
CI: Confidence interval
EAEC: Enteroaggregative *E. coli*
EE: Environmental enteropathy
EED: Environmental enteric dysfunction
*E. coli*: *Escherichia Coli*
EIEC: Enteroinvasive *E. coli*
ELISA: Enzyme-linked immunosorbent assays
EPEC: Enteropathogenic *E. coli*
ETEC: Enterotoxigenic *E. coli*
IGF-1: Insulin-like growth factor 1
L: M: Lactulose: mannitol
LMICs: Low and middle income countries
MPO: Myeloperoxidase
NEO: Neopterin
ng/ml: Nanogram per milliliter
nmol/l: Nanomoles per milliliter
OD: Optical density
SD: Standard deviation
WASH: Water, sanitation and hygiene
